# Combined effects of nutrition, inflammatory status, and sleep quality on mortality in cancer survivors

**DOI:** 10.1186/s12885-024-13181-x

**Published:** 2024-11-27

**Authors:** Tingyu Zhao, Hui Zhao, Xiao Zhang, Xingyu Jiang, Qi Liang, Siqi Ni, Yi Jiao, Jiamei Yu, Jianghong Dai, Mulong Du, Lingxiang Liu

**Affiliations:** 1 Department of Oncology, The First Affiliated Hospital of Nanjing Medical University, 300 Guangzhou Road, Nanjing, 210029 China; 2 Department of Epidemiology and Biostatistics, School of Public Health, Xinjiang Medical University, 393, Xinyi Road, Xinshi District, Urumqi, 830000 China; 3 Department of Environmental Genomics, Jiangsu Key Laboratory of Cancer Biomarkers, Prevention and Treatment, Collaborative Innovation Center for Cancer Personalized Medicine, School of Public Health, Nanjing Medical University, 101 Longmian Avenue, Jiangning District, Nanjing, 211166 China; 4 Department of Biostatistics, Center for Global Health, School of Public Health, Nanjing Medical University, 101 Longmian Avenue, Jiangning District, Nanjing, 211166 China

**Keywords:** Cancer mortality, Advanced lung cancer inflammatory index, Sleep quality, Patient care

## Abstract

**Background:**

Cancer survivors face many challenges in long-term health management, including malnutrition, systemic inflammation, and sleep issues, which significantly affect their survival and quality of life.

**Methods:**

A prospective cohort study was derived from the National Health and Nutrition Examination Survey from 2005–2018 harboring 1,908 cancer survivors (weighted population, 11,453,293), of whom 688 deaths (220 from cancer mortality, 468 from non-cancer mortality). The Advanced Lung Cancer Inflammation Index (ALI) was used as a measure of nutritional status and systemic inflammation in cancer patients. Weighted multivariable Cox proportional hazards regression models were utilized to explore the independent and combined effects of ALI and sleep quality on mortality outcomes.

**Results:**

The participants with a high ALI were more likely to be female, aged 40 to 64 years, non-Hispanic white, and have a higher BMI. We observed that elevated ALI levels were associated with decreased risks of all-cause mortality (Hazard ratio [HR] = 0.601, 95% Confidence interval [CI] = 0.521–0.695, *P* < 0.001), cancer-specific mortality (HR = 0.659, 95% CI = 0.497–0.870, *P* = 3.34 × 10^–3^) and non-cancer-specific mortality (HR = 0.579, 95% CI = 0.478–0.701, *P* < 0.001). Similarly, better sleep quality (e.g., without sleep troubles) was associated with lower risks of all-cause mortality (HR = 0.761, 95% CI = 0.620–0.933, *P* = 8.79 × 10^–3^) and non-cancer-specific mortality (HR = 0.713, 95% CI = 0.572–0.890, *P* = 2.80 × 10^–3^). Notably, the joint analysis showed that cancer survivors with higher ALI levels and better sleep quality (e.g., standard sleep duration) had the lowest risks of all-cause (HR = 0.468, 95% CI = 0.352–0.622, *P* < 0.001), cancer-specific mortality (HR = 0.631, 95% CI = 0.333–0.672, *P* = 7.59 × 10^–3^) and non-cancer-specific mortality (HR = 0.440, 95% CI = 0.315–0.615, *P* < 0.001).

**Conclusions:**

This study suggests that better nutritional and inflammatory status, combined with good sleep quality, may contribute to improved survival among cancer survivors. These results underscore the potential clinical importance of integrating nutritional and sleep quality assessments into the long-term care of cancer survivors to enhance their overall prognosis.

**Supplementary Information:**

The online version contains supplementary material available at 10.1186/s12885-024-13181-x.

## Introduction

With the continuous advancement of medical technology, the number of cancer patients has significantly increased, and the overall survival rates of these patients have improved significantly.”. For instance, the 5-year survival rate for all cancers combined has improved from 49% in the 1970s to 69% in recent years, largely due to advancements in early detection and treatment modalities [1]. However, cancer patients face a multitude of challenges in their long-term health management, including but not limited to malnutrition [2], persistent inflammatory states [3], and sleep quality issues [4], which significantly impact both their quality of life and overall survival outcomes.

Systemic inflammatory responses, including changes in biomarkers such as C-reactive protein (CRP), neutrophil-to-lymphocyte ratio (NLR), and platelet-to-lymphocyte ratio (PLR), have been shown to be closely linked with the prognosis of various cancer types [5, 6]. Nutritional status also has a significant influence on the prognosis of cancer patients, with the prognostic nutritional index (PNI) considered an important factor affecting the survival rate, disease progression, and treatment response of cancer patients [7, 8]. Inflammatory status can independently predict the survival rate of cancer patients. Similarly, nutritional status also serves as a significant predictor of survival [9]. Previous studies have shown that considering both inflammation and nutritional indicators can more accurately predict the prognosis of cancer patients [10, 11]. The advanced lung cancer inflammation index (ALI) acts as an integrated marker for assessing a patient's nutritional status and systemic inflammation, calculated using the body mass index (BMI), serum albumin (Alb) concentrations, and the NLR. ALI was originally designed to predict the survival outcomes of patients with metastatic non-small cell lung cancer (NSCLC) [12]. While the adoption of ALI in clinical practice remains limited, it has demonstrated significant prognostic value in various research settings. It had demonstrated significant prognostic value (low ALI was associated with poor overall survival [OS]) in a variety of cancer types, including small cell lung cancer, gastric cancer, colon cancer, head and neck squamous cell carcinoma, and diffuse large B-cell lymphoma [13, 14]. Therefore, as an integrated indicator that reflects the nutritional status and systemic inflammation of patients, the ALI holds significant importance for evaluating cancer patients' long-term prognosis and developing personalized treatment plans.

Sleep problems are exceedingly common in modern society, affecting individuals in the general population across various ages, genders, and occupations [15–17]. Poor sleep quality was linked not only to a heightened risk of cancer development, but also to impacts on cancer patients’ survival duration and quality of life [18–20]. In individuals with advanced cancer, a curvilinear association had been noted between sleep duration and mortality, and both shorter and longer sleep durations are connected to higher mortality rates [18]. Reduced sleep in cancer patients was associated with elevated levels of CRP and interleukin-6 (IL-6), leading to an increase in the body's inflammatory levels [21, 22]. Furthermore, poor sleep quality was significantly associated with elevated risk of malnutrition (sleeping less than 6 h and more than 9 h increased the chances of malnutrition) in cancer patients [19]. However, the complex associations between sleep quality, nutritional status, and inflammation levels with the prognosis of cancer patients remain to be clarified.

Malnutrition can exacerbate the side effects of treatment, reduce tolerance to therapies, and negatively affect recovery [7, 8]. Persistent inflammation has been closely linked to cancer progression and recurrence, making it a critical factor in long-term prognosis [5]. Sleep disturbances, common in cancer patients, are associated with poor mental and physical recovery, further compounding the negative outcomes [20]. Therefore, this study focuses on these three key outcomes as they represent major, modifiable factors that can potentially improve the overall health and survival of cancer patients. Addressing these issues provides an opportunity to enhance patient care and long-term management.

This study aimed to investigate the impact of the ALI and sleep quality on the long-term health and survival rates of cancer patients,with the goal of identifying key factors affecting long-term health management and survival to inform future interventions. Through a comprehensive assessment and analysis of these key factors, we hope to provide more personalized and effective treatment and care guidance for cancer patients, thereby promoting their overall well-being and long-term survival.

## Methods

### Study population

This prospective cohort study made use of a nationally representative sample from the National Health and Nutrition Examination Survey (NHANES). The Centers for Disease Control and Prevention (CDC) ascertained causes and time to death information by linking eligible NHANES participants to the National Death Index. The study, which combines questionnaire surveys, laboratory examinations and physical examinations, has been conducted biennially since 1999, focusing on various population groups or health issues. The Institutional Review Board of the National Center for Health Statistics granted approval for the NHANES study, with participants giving their written informed consent.

Initially, a total of 3,782 cancer survivors were recruited across seven 2-year NHANES survey cycles from 2005–2006, 2007–2008, 2009–2010, 2011–2012, 2013–2014, 2015–2016, and 2017–2018. Participants were queried, "Have you ever been told by a doctor or other health professional that you had cancer or a malignancy of any kind?" Those who answered affirmatively were defined as cancer survivors from diagnosis. From these participants, individuals under the age of 40 (*n* = 252), missing ALI-related data (*n* = 539), lacking sleep quality data (*n* = 915), or missing any covariate-related information (*n* = 168) were excluded from the analysis. The final analysis included 1908 patients (eFigure 1). The count of cancer survivors, segmented by gender and type of cancer, is provided in eTable 1.

### Assessment of ALI and sleep quality

The ALI was calculated with the formula: ALI = BMI × Alb/NLR, where BMI was determined by dividing weight in kilograms by the square of height in meters, Alb stood for serum albumin levels measured in grams per deciliter, and NLR was the ratio of absolute neutrophil count to absolute lymphocyte count. ALI was categorized into two groups: Low (≤ 53.2) and High (> 53.2).

Sleep quality-related data encompassed sleep duration, trouble sleeping, and sleep disorders [21]. Sleep duration was defined by the response to the question, "On workdays or school days, how many hours of sleep do you usually get at night?" Participants were queried, "Have you ever told a doctor or other health professional that you have trouble sleeping?" Those who answered "yes" were considered to have trouble sleeping. Additionally, participants were queried, "Have you ever been told by a doctor or other health professional that you have a sleep disorder?" Those who answered "yes" were regarded as having a sleep disorder.

To explore the potential mediating role of ALI and sleep duration on all-cause mortality, we performed a mediating analysis, focusing on the role of BMI and NLR. Use the Mediation package in R software version 4.3.1 for causal step analysis.

### Covariate definitions

In this study, we considered the following variables as covariates: gender, age, race/ethnicity, BMI, family poverty income ratio, level of education and age at first cancer diagnosis. The categories used to classify race/ethnicity were non-Hispanic white, non-Hispanic black, other Hispanic, Mexican American, and other race/ethnicity. BMI was divided into three groups: < 25, 25.0 to 29.9, and ≥ 30. The family poverty income ratio was categorized into three groups: < 1.3, 1.3 to ≤ 3.5, and ≥ 3.5. For educational levels, the categories were less than high school (ninth grade or less, ninth to eleventh grade including twelfth grade without a diploma), high school (high school diploma or General Equivalency Diploma, some college or associate's degree), and beyond high school (college graduate or higher). In addition, we grouped cancer types into digestive, genitourinary, skin, breast, and other cancers to assess the association of ALI levels and sleep quality with mortality in different cancer types.

### Mortality definitions

The primary outcome of this study was centered on all-cause mortality, with secondary outcomes addressing mortality specific to cancer and mortality unrelated to cancer. All-cause mortality encompassed deaths from any cause, cancer-specific mortality was determined by the UCOD_LEADING codes for Malignant neoplasms (019–043), and deaths from causes other than cancer were categorized as non-cancer-specific mortality.

### Statistical analysis

Adhering to the NHANES analysis guidelines, this study integrated sample weights, clustering, and stratification in its analyses to precisely estimate variance and guarantee that the findings are nationally representative of cancer survivors in the United States. Baseline characteristics provided insights into different levels of the ALI and the quality of sleep. Restricted cubic splines (RCS) were used to illustrate the non-linear relationship between ALI or sleep duration and the HR for all-cause and non-cancer-specific mortality. We calculated the best cutoff value for ALI on the ROC curve, corresponding to the point with the highest Youden index, where both sensitivity (true positive rate) and specificity (1-false positive rate) reach their maximum simultaneously. To assess the independent association between ALI and sleep quality with all-cause, cancer-specific, and non-cancer mortality, weighted multivariable Cox proportional hazards regression models were used to calculate Hazard ratio (HR) and 95% Confidence interval (CI). The final multivariable models were adjusted for factors including gender, age, race and ethnicity, BMI, family poverty income ratio, education level, and age at first cancer diagnosis. To evaluate the combined impact of ALI and sleep quality on mortality risk, participants were categorized based on ALI and sleep quality, and weighted multivariable Cox proportional hazards regression models adjusted for the same set of covariates were utilized. In the analysis of cancer-specific and non-cancer-specific mortality, it is considered that both short and long sleep durations were associated with an increased risk of mortality. Sleep duration was simplified into non-standard sleep duration (< 7 & > 9 h) and standard sleep duration (7 to 9 h). All analyses were conducted using a two-sided approach, with an alpha significance level set at 0.05. All statistical analyses were performed using R version 4.3.1.

## Results

### Baseline characteristics

A total of 1,908 cancer survivors were included in the study (49% male; weighted population: 11,453,293). During the follow-up period (median, 8.1 years), there were 688 deaths, including 220 from cancer and 468 from non-cancer-specific causes. To stratify the ALI level, we used ROC curve analysis to balance the sensitivity and specificity and determined the optimal cutoff value of ALI to be 53.2 (eFigure 2); accordingly, patients were classified into two groups as low (ALI ≤ 53.2, *n* = 951) and high (ALI > 53.2, *n* = 957). Participants who were female, aged between 40 to 64 years, non-Hispanic white, and higher BMI were more likely to have a high ALI (Table 1).

**Table 1:** Baseline characteristics of US cancer survivors according to the advanced lung cancer inflammation index

Characteristic	All	Weighted	No. of participants by ALI		*P*-Value	Weighted-P
			Low	High		
Overall	1,908	11,453,293	(*N* = 951)	(*N* = 957)		
Gender (%)					< 0.001	6.17 × 10^–2^
Male	935(49.0)	6,291,139(54.9)	519(54.6)	416(43.5)		
Female	973(51.0)	5,162,145(45.1)	432(45.4)	541(56.5)		
Age(years) (%)					< 0.001	< 0.001
40 to 64	698(36.6)	5,452,953(47.6)	253(26.6)	445(46.5)		
≥ 65	1,210(63.4)	6,000,340(52.4)	698(73.4)	512(53.5)		
Race and ethnicity (%)					< 0.001	2.36 × 10^–3^
Non-Hispanic White	1,376(72.1)	10,115,222(88.3)	731(76.9)	645(67.4)		
Non-Hispanic Black	267(14.0)	555,419(4.8)	98(10.3)	169(17.7)		
Mexican American	112(5.9)	219,320(1.9)	47(4.9)	65(6.8)		
Other	153(8.0)	563,331(4.9)	75(7.9)	78(8.2)		
Weight status, BMI (%)					< 0.001	< 0.001
< 25	537(28.1)	3,371,971(29.4)	359(37.7)	178(18.6)		
25 to < 30	682(35.7)	4,080,994(35.6)	369(38.8)	313(32.7)		
≥ 30	689(36.7)	4,000,328(34.9)	223(23.5)	466(48.7)		
Family poverty income ratio (%)					9.09 × 10^–2^	1.40 × 10^–1^
< 1.3	420(22.0)	1,503,362(13.1)	208(21.9)	212(22.2)		
1.3 to < 3.5	789(41.4)	4,236,240(37)	415(43.6)	374(39.1)		
≥ 3.5	699(36.6)	5,713,690(4939)	328(34.5)	371(38.8)		
Educational attainment (%)					8.41 × 10^–1^	5.21 × 10^–1^
< High school	428(22.4)	1,580,204(13.8)	214(22.5)	214(22.4)		
High school	434(22.7)	2,420,687(21.1)	211(22.2)	223(23.3)		
> High school	1,046(54.8)	7,452,401(65.1)	526(55.3)	520(54.3)		
Age at cancer first diagnosed (years) (%)					< 0.001	< 0.001
< 40	291(15.3)	2,243,055(19.6)	108(11.4)	183(19.1)		
40 to 60	810(42.5)	5,451,222(47.6)	368(38.7)	442(46.2)		
> 60	807(42.3)	3,759,016(32.8)	475(49.9)	332(34.7)		
Sleep duration(hours) (%)					< 0.001	< 0.001
< 7	652(34.2)	3,498,066(30.5)	300(31.5)	352(36.8)		
7 to 9	1,183(62.0)	7,606,071(66.4)	601(63.2)	582(60.8)		
> 9	73(3.8)	349,156(3)	50(5.3)	23(2.4)		
Sleep trouble (%)					3.83 × 10^–2^	2.44 × 10^–2^
Yes	654(34.3)	7,274,997(63.5)	304(32.0)	350(36.6)		
No	1,254(65.7)	4,178,295(36.5)	647(68.0)	607(63.4)		
Sleep disorder (%)					< 0.001	1.02 × 10^–2^
Yes	242(12.7)	1,661,214(14.5)	93(9.8)	149(15.6)		
No	1,666(87.3)	9,792,079(85.5)	858(90.2)	808(84.4)		

*Abbreviations*: *ALI* Advanced lung cancer inflammation index, *BMI* Body mass index

### Relationship between ALI or sleep duration and mortality outcomes

**Fig. 1:**
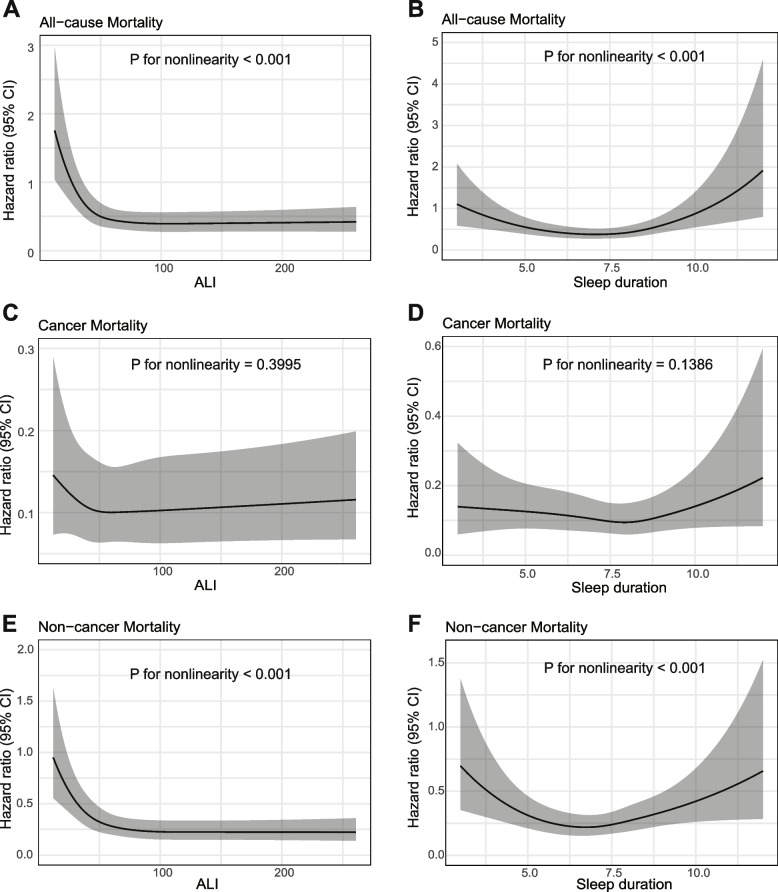
Association between advanced lung cancer inflammation index or sleep duration with all-cause, cancer and non-cancer mortality among cancer survivors. **A-B** All-cause mortality; **C-D** Cancer mortality; (**E**-**F**) Non-cancer mortality

Figure 1 illustrated, after controlling for a range of potential confounders, that restricted cubic splines (RCS) revealed an L-shaped curve relationship between ALI and both all-cause and non-cancer-specific mortality rates. As ALI values increase, there was a marked reduction in the rates of all-cause and non-cancer-specific mortality (P for non-linearity < 0.0001). Similarly, sleep duration was also significantly associated with the all-cause mortality and non-cancer-specific mortality, displaying a U-shaped curve (*P* for non-linearity < 0.0001). With increasing sleep duration, we observed a significant U-shaped association with all-cause and non-cancer-specific mortality, where mortality rates initially decrease and then rise.

The mediation effect analysis results indicated that ALI does not play a significant mediating role between sleep duration and all-cause mortality, whereas BMI (IE = 0.0023, *P* < 0.001) and NLR (IE = 0.0031, *P* = 0.028) have significant mediating effects (eFigure3).

### Association between ALI or sleep quality and mortality outcomes

Elevated ALI levels were linked to lower risks of all-cause and non-cancer-specific mortality in cancer survivors, exhibiting HR of 0.601 (95%CI = 0.521–0.695, *P* < 0.001) for all-cause mortality, 0.658 (95%CI = 0.497–0.870, *P* = 3.34 × 10^–3^) for cancer-specific mortality and 0.579 (95%CI = 0.478–0.701, *P* < 0.001) for non-cancer-specific mortality, as detailed in Table 2. Additionally, patients experiencing sleep durations of less than 7 h (HR = 1.315, 95%CI = 1.059–1.634, *P* = 1.34 × 10^–2^) or more than 9 h (HR = 1.919, 95%CI = 1.308–2.816, *P* < 0.001) faced increased risks of all-cause mortality as well compared to survivors whose sleep durations fell within the 7 to 9 h range (Table 2). Besides, survivors without sleep troubles exhibited reduced risks of all-cause (HR = 0.761, 95%CI = 0.620–0.933, *P* = 8.79 × 10^–3^) and non-cancer-specific (HR = 0.713, 95%CI = 0.572–0.890, *P* = 2.80 × 10^–3^) mortality in comparison to those encountering sleep troubles (Table 2).

**Table 2:** Association of advanced lung cancer inflammation index and sleep quality with mortality among US cancer survivors age40 years or older, NHANES, 2005 to 2018

Mortality outcome	Death/No.	Weighted death (%)	Hazard ratio (95 % CI)			
			Model 1^a^	P	Model 2^b^	P
**All-cause**						
ALI						
Low	440/951	1,960,259(17.1)	1		1	
High	248/957	1,086,135(9.5)	**0.706(0.593-0.840)**	**<0.001**	**0.601(0.521-0.695)**	**<0.001**
Sleep duration (hours)						
<7	233/652	967,565(8.4)	**1.365(1.111-1.677)**	**3.04×10**^-3^	**1.315(1.059-1.634)**	**1.34×10**^-2^
7 to 9	406/1,183	1,862,277(16.3)	1		1	
>9	49/73	216,552(1.9)	**1.811(1.256-2.610)**	**1.47×10**^-3^	**1.919(1.308-2.816)**	**<0.001**
Sleep trouble						
Yes	228/654	1,071,980(9.4)	1		1	
No	460/1,254	1,974,414(17.2)	**0.790(0.630-0.992)**	**4.20×10**^-2^	**0.761(0.620-0.933)**	**8.79×10**^-3^
Sleep disorder						
Yes	76/242	2,669,872(23.3)	1		1	
No	612/1,666	376,522(3.3)	0.876(0.627-1.224)	4.38×10^-1^	0.739(0.510-1.070)	1.10×10^-1^
**Cancer**						
ALI						
Low	126/951	603,317(5.3)	1		1	
High	94/957	392,233(3.4)	**0.745(0.561-0.990)**	**4.21×10**^-2^	**0.658(0.497-0.870)**	**3.34×10**^-3^
Sleep duration (hours)						
<7	78/652	316,380(2.8)	1.280(0.917-1.786)	1.46×10^-1^	1.193(0.846-1.683)	4.45×10^-1^
7 to 9	128/1,183	619,319(5.4)	1		1	
>9	14/73	59,852(0.5)	1.714(0.892-3.293)	1.06×10^-1^	1.688(0.832-3.426)	1.08×10^-1^
Sleep trouble						
Yes	75/654	334,166(2.9)	1		1	
No	145/1,254	661,384(5.8)	0.880(0.600-1.293)	5.16×10^-1^	0.869(0.603-1.254)	4.30×10^-1^
Sleep disorder						
Yes	27/242	118,632(1)	1		1	
No	193/1,666	876,918(7.7)	1.069(0.600-1.905)	8.21×10^-1^	1.027(0.582-1.814)	8.59×10^-1^
**Non-cancer**						
ALI						
Low	314/951	1,356,943(11.8)	1		1	
High	154/957	693,901(6.1)	**0.690(0.556-0.856)**	**<0.001**	**0.579(0.478-0.701)**	**<0.001**
Sleep duration (hours)						
<7	155/652	651,186(5.7)	**1.412(1.126-1.770)**	**2.80×10**^-3^	**1.383(1.094-1.749)**	**6.70×10**^-3^
7 to 9	278/1,183	1,242,958(10.9)	1		1	
>9	35/73	156,700(1.4)	**1.841(1.221-2.774)**	**3.57×10**^-3^	**2.009(1.303-3.098)**	**1.59×10**^-3^
Sleep trouble						
Yes	153/654	737,814(6.4)	1		1	
No	315/1,254	1,313,030(11.5)	**0.750(0.583-0.964)**	**2.48×10**^-2^	**0.713(0.572-0.890)**	**2.80×10**^-3^
Sleep disorder						
Yes	49/242	257,890(2.3)	1		1	
No	419/1,666	1,792,954(15.7)	0.777(0.533-1.133)	1.90×10^-1^	**0.634(0.411-0.979)**	**4.00×10**^-2^

*Abbreviations*: *ALI* Advanced lung cancer inflammation index, *NHANES* The National Health and Nutrition Examination Survey

^a^Adjusted for age and gender (male/female)

^b^Multivariable model additionally adjusted for race and ethnicity (non-Hispanic Black, Hispanic, non-Hispanic White, other), educational attainment (<high school, high school, >high school), family poverty income ratio (<1.3, 1.3 to ≤ 3.5, ≥3.5) and age of cancer diagnosis.

As described in eTable 2, the relationship between sleep troubles and mortality was more consistent among male survivors than among female survivors. Standard sleep duration was associated with reduced mortality in patients aged 40 to 64, but not in patients aged 65 and older. In addition, mortality was more strongly associated with sleep quality in overweight and obese patients and in patients aged 40 to 60 years at first diagnosis of cancer. Further in the stratification analysis by cancer types (eTable 3), high levels of ALI reduced the risk of death by approximately 46% for digestive cancer survivors, 37% for genitourinary cancer survivors, and 45% for skin cancer survivors. Digestive cancer survivors without sleep troubles had lower all-cause mortality (HR = 0.531, 95%CI = 0.301–0.938, *P* = 2.90 × 10^–2^), as well as skin cancers with standard sleep duration (HR = 0.563, 95%CI = 0.416–0.762, *P* < 0.001).

### Joint effect of ALI and sleep quality on mortality

**Fig. 2:**
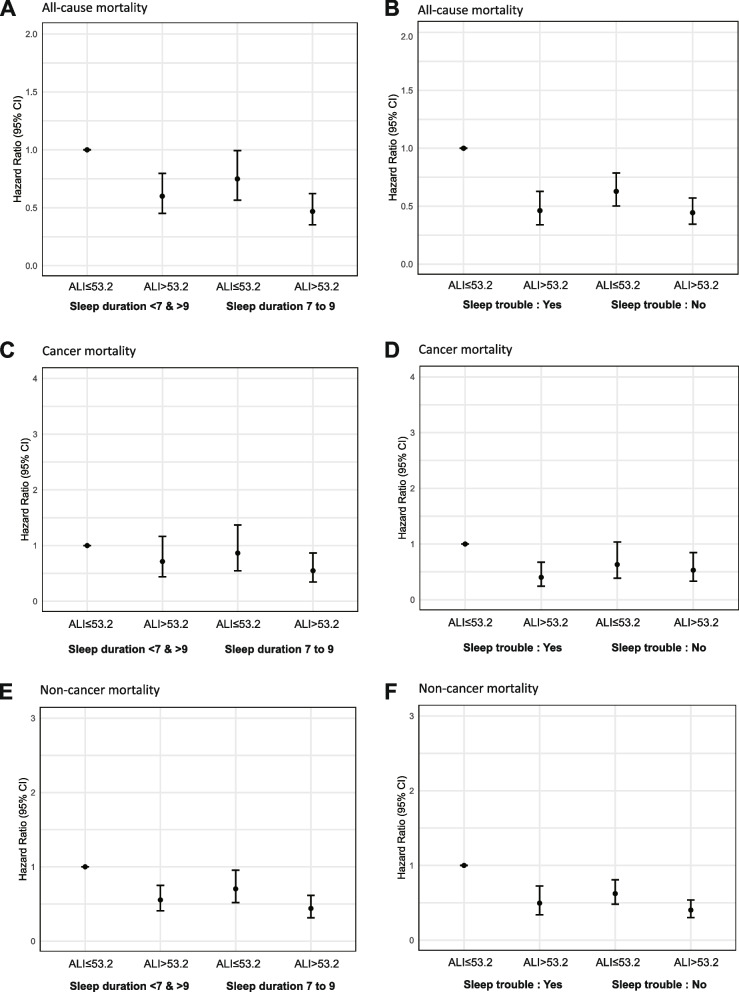
Joint association of advanced lung cancer inflammatory index and sleep quality with all-cause, cancer, and non-cancer mortality among cancer survivors. **A-B** All-cause mortality; **C-D** Cancer mortality; **E-F** Non-cancer mortality

In the joint analysis, the combinations of higher ALI levels with standard sleep duration and higher ALI levels without sleep problems were all beneficially associated with lower risks of mortality (Fig. 2 and eTable 4). Specifically, compared to survivors with non-standard sleep duration and low ALI levels, those with standard sleep duration and high ALI levels had the lowest risk of all-cause mortality (HR = 0.468, 95% CI = 0.352–0.622, *P* < 0.001). Similar association were observed for cancer-specific mortality (HR = 0.865, 95% CI = 0.347–0.867, *P* = 1.02 × 10^–2^) and non-cancer-specific mortality (HR = 0.440, 95% CI = 0.315–0.615, *P* < 0.001). As well, survivors with high ALI levels and no sleep troubles had the lowest risk of all-cause mortality (HR = 0.444, 95%CI = 0.345–0.570, *P* < 0.001), cancer-specific mortality (HR = 0.531, 95%CI = 0.333–0.672, *P* = 7.59 × 10^–3^) and non-cancer-specific mortality (HR = 0.403, 95%CI = 0.303–0.537, *P* < 0.001) compared to survivors with low ALI levels and sleep troubles.

## Discussion

This study explored the effects of the ALI and sleep quality on the longevity and health outcomes of cancer survivors. The results indicated that ALI levels, sleep duration, and the presence of sleep troubles are independent factors predicting all-cause and non-cancer-specific mortality in cancer survivors. Specifically, survivors with higher ALI levels exhibited a hazard ratio (HR) for all-cause mortality of 0.601 (95% CI: 0.521–0.695), suggesting a nearly 40% reduction in the risk of death, which may represent significant survival benefits in a clinical context. Similarly, standard sleep duration (7 to 9 h) was associated with a comparable reduction in mortality risk (HR for all-cause mortality = 1.315), indicating that deviations from this duration may increase mortality risk. Notably, survivors without sleep troubles had a lower hazard ratio for all-cause mortality (HR = 0.761, 95% CI: 0.620–0.933), highlighting the potential importance of preventing and alleviating sleep disturbances in the management of sleep quality. These findings underscore the potential for improving survival outcomes through the enhancement of ALI levels and sleep quality, which could be highly valuable in personalized health management.

Previous studies had established that both inflammatory and nutritional statuses are associated with sleep quality [21, 22]. Inflammatory markers served as mediating factors in the relationship between sleep duration and mortality, where higher levels of inflammation are linked with insufficient sleep [22]. Among elderly patients, non-standard sleep durations increased the risk of malnutrition, and as the risk of malnutrition rises, there is a concurrent decline in sleep quality [23]. Sleep and nutritional status may further influence cancer progression and patient survival by affecting the function of the hypothalamic–pituitary–adrenal (HPA) axis and autonomic nervous system. Overactivation of the HPA axis may lead to chronic inflammation and immunosuppression, which promotes the growth of cancer cells. We also observed associations suggesting that inflammatory and nutritional statuses may act as mediating factors between sleep duration and mortality. By investigating these correlations, our study offers valuable insights into factors that may influence long-term health outcomes for these patients.

The nutritional status and inflammatory response of cancer survivors were closely related to their prognosis, and both are considered independent prognostic factors [24]. Poorer nutritional status led to higher medium-term and long-term mortality, reduced likelihood of completing cancer treatment, and increased medical consumption [25]. BMI, a common indicator for assessing nutritional status, was also significantly associated with morbidity and mortality for many types of cancer [24, 26]. In addition, as an indicator of the nutritional status of cancer survivors, albumin level also had important prognostic significance in predicting the survival rate of cancer survivors. NLR is an effective biomarker for evaluating systemic inflammatory response and can be used to predict inflammatory status and prognosis of a variety of diseases, especially in cancer with significant clinical effects [27]. Studies had shown that combining nutritional and inflammatory measures can provide more accurate information about cancer prognosis than either measure alone [28, 29]. Hence, the development of a composite index encompassing both nutrition and inflammation is crucial for evaluating the prognosis of cancer survivors. The ALI score provided a holistic assessment of systemic health by integrating nutritional and inflammatory elements, establishing it as a superior predictive marker for cancer prognosis compared to other indices [30]. In this study, ALI was demonstrated to be significantly associated with the prognosis of cancer survivors, underscoring its utility in predicting outcomes and guiding treatment strategies.

Poor sleep quality is prevalent among cancer survivors [31, 32], potentially due to a variety of factors such as pain, depression, and a sense of hopelessness, and may persist throughout different stages of cancer treatment [20, 33]. In our study, we found that sleep duration and sleep disturbances are independent prognostic factors for both all-cause mortality and non-cancer-specific mortality among cancer survivors. Notably, the impact of sleep disturbances on all-cause mortality exhibited gender differences, with stronger and more consistent associations observed among male survivors. Previous research has shown that sleep disorders are more common in women, particularly during the perimenopausal and postmenopausal periods, likely due to fluctuations in gonadal hormones, which may partly explain these gender differences [34–36]. Additionally, our findings suggest that the relationship between sleep duration and all-cause mortality varies by age, with a more pronounced association in patients aged 40 to 64 years. This may be attributed to the higher likelihood of short sleep duration reported by patients aged 65 or older compared to their younger counterparts [37]. These results underscore the importance of regularly assessing and addressing sleep quality in the management of cancer survivors, considering the critical role that sleep plays in their overall prognosis. Furthermore, they highlight the need for personalized interventions that take into account individual differences in gender and age.

While our study provides valuable insights into the combined effects of nutritional status, inflammatory markers, and sleep quality on mortality in cancer survivors, several limitations should be acknowledged: The study’s observational nature limits the ability to establish causality between the observed associations. Despite adjusting for multiple potential confounding factors, the lack of data on cancer treatment and stage at diagnosis, along with other unidentified or unmeasurable variables, may still affect the interpretation of the results. Sleep quality and duration were assessed using self-reported questionnaires, which may be subject to recall bias and misclassification. Objective measurements such as actigraphy or polysomnography were not utilized, which could have provided more accurate assessments of sleep patterns. Moreover, although attempts were made to conduct a detailed analysis based on the type and location of cancer, further exploration is needed to understand how specific types of cancer affect the relationship between ALI or sleep and mortality rates.

This study suggests that improved nutritional and inflammatory status, optimal sleep duration, and the absence of sleep troubles may be associated with lower risks of all-cause and non-cancer-specific mortality among cancer survivors. These findings highlight key factors that may influence the long-term health and survival of cancer survivors. Although our study did not assess specific interventions, the identified associations suggest potential targets for future research to explore how interventions addressing these factors might improve outcomes. Further studies are needed to determine the efficacy of nutritional, inflammatory, and sleep-related interventions in improving the prognosis of cancer survivors.

## Supplementary Information

Supplementary Material 1.

Supplementary Material 2.

Supplementary Material 3.

Supplementary Material 4.

## Data Availability

All data that support the findings of this study can be queried in https://figshare.com/articles/dataset/NHANES_1988-2018/21743372 and https://www.cdc.gov/nchs/nhanes/index.htm.
