# Supplementary material for: Combined effects of nutrition, inflammatory status, and sleep quality on mortality in cancer survivors
Source: BMC Cancer. 2024 Nov 27;24:1456. doi: 10.1186/s12885-024-13181-x (PMC11600600; doi:10.1186/s12885-024-13181-x)
Supplement: Supplementary file 1 — Supplementary Material 1. [file 12885_2024_13181_MOESM1_ESM.docx]

| **eTable 1. Numbers of cancer survivors by cancer type and gender, NHANES 2005-2018.** | | | | |
| --- | --- | --- | --- | --- |
| **Cancer type** | **Group** | **Numbers of cancer survivors** | | |
|  |  | **Total** | **Male** | **Female** |
| **Overall** |  | 1908 | 935 | 973 |
| Bladder | Genitourinary cancer | 33 | 24 | 9 |
| Blood | Other cancer | 3 | 3 | 0 |
| Bone | Other cancer | 6 | 4 | 2 |
| Brain | Other cancer | 11 | 7 | 4 |
| Breast | Breast cancer | 307 | 0 | 307 |
| Cervix(cervical) | Genitourinary cancer | 110 | 0 | 110 |
| Colon | Digestive cancer | 124 | 66 | 58 |
| Esophagus(esophageal) | Digestive cancer | 8 | 7 | 1 |
| Gallbladder | Digestive cancer | 1 | 0 | 1 |
| Kidney | Genitourinary cancer | 34 | 22 | 12 |
| Larynx/windpipe | Other cancer | 10 | 9 | 1 |
| Leukemia | Other cancer | 13 | 11 | 2 |
| Liver | Digestive cancer | 6 | 3 | 3 |
| Lung | Other cancer | 41 | 26 | 15 |
| Lymphoma/Hodgkin's disease | Other cancer | 33 | 17 | 16 |
| Melanoma | Skin cancer | 111 | 59 | 52 |
| Mouth/tongue/lip | Other cancer | 11 | 7 | 4 |
| Nervous system | Other cancer | 0 | 0 | 0 |
| Ovary(ovarian) | Genitourinary cancer | 34 | 0 | 34 |
| Pancreas(pancreatic) | Digestive cancer | 4 | 3 | 1 |
| Prostate | Genitourinary cancer | 301 | 301 | 0 |
| Rectum(rectal) | Digestive cancer | 7 | 2 | 5 |
| Skin(non-melanoma) | Skin cancer | 331 | 192 | 139 |
| Skin(don't know what kind) | Skin cancer | 152 | 101 | 51 |
| Soft tissue(muscle or fat) | Other cancer | 5 | 3 | 2 |
| Stomach | Digestive cancer | 11 | 7 | 4 |
| Testis(testicular) | Genitourinary cancer | 7 | 7 | 0 |
| Thyroid | Other cancer | 36 | 8 | 28 |
| Uterus(uterine) | Genitourinary cancer | 67 | 0 | 67 |
| Other | Other cancer | 91 | 46 | 45 |
| Abbreviations: NHANES, the National Health and Nutrition Examination Survey. | | | | |

| **eTable 2. Stratified analysis of advanced lung cancer inflammation index and sleep quality with all-cause mortality among US cancer survivors age40 years or older, NHANES, 2005 to 2018.** | | | | | | |
| --- | --- | --- | --- | --- | --- | --- |
| **Mortality outcome** | **Death/No.** | **Weighted death(%)** | **Hazard ratio(95 % CI)** | | |  |
|  |  |  | **Model 1^a^** | **P** | **Model 2^b^** | **P** |
| **Male** |  |  |  |  |  |  |
| ALI |  |  |  |  |  |  |
| Low | 276/517 | 11047438(21.5) | 1 | 1.75×10^-3^ | 1 | **2.70×10^-6^** |
| High | 128/411 | 523126(10.2) | 0.680(0.530-0.873) |  | 0.567(0.447-0.719) |  |
| Sleep duration(hours) |  |  |  |  |  |  |
| <7 & >9 | 166/346 | 618634(12) | 1 | 1.19×10^-3^ | 1 | 1.09×10^-1^ |
| 7 to 9 | 240/582 | 1011931(19.6) | 0.641(0.490-0.838) |  | 0.752(0.530-1.066) |  |
| Sleep trouble |  |  |  |  |  |  |
| Yes | 130/271 | 588139(11.4) | 1 | 2.31×10^-4^ | 1 | **5.05×10^-6^** |
| No | 374/657 | 1042426(20.2) | 0.599(0.455-0.787) |  | 0.537(0.411-0.701) |  |
| **Female** |  |  |  |  |  |  |
| ALI |  |  |  |  |  |  |
| Low | 162/443 | 848821(13.5) | 1 | 2.37×10^-2^ | 1 | **1.48×10^-3^** |
| High | 118/538 | 560278(8.9) | 0.733(0.564-0.954) |  | 0.680(0.537-0.863) |  |
| Sleep duration(hours) |  |  |  |  |  |  |
| <7 & >9 | 116/376 | 563075(9) | 1 | 1.06×10^-1^ | 1 | 8.58×10^-2^ |
| 7 to 9 | 164/595 | 846024(13.5) | 0.796(0.598-1.059) |  | 0.794(0.610-1.033) |  |
| Sleep trouble |  |  |  |  |  |  |
| Yes | 96/379 | 481398(7.7) | 1 | 7.15×10^-1^ | 1 | 6.70×10^-1^ |
| No | 184/592 | 927701(14.8) | 1.052(0.741-1.494) |  | 1.069(0.786-1.456) |  |
|  |  |  |  |  |  |  |
| **Age 40 to 64** |  |  |  |  |  |  |
| ALI |  |  |  |  |  |  |
| Low | 51/253 | 250247(4.6) | 1 | 6.36×10^-2^ | 1 | **3.21×10^-2^** |
| High | 52/445 | 251726(4.6) | 0.591(0.339-1.030) |  | 0.585(0.358-0.955) |  |
| Sleep duration(hours) |  |  |  |  |  |  |
| <7 & >9 | 65/310 | 209444(3.8) | 1 | 5.09×10^-5^ | 1 | **2.40×10^-2^** |
| 7 to 9 | 38/388 | 292529(5.4) | 0.363(0.223-0.593) |  | 0.560(0.339-0.927) |  |
| Sleep trouble |  |  |  |  |  |  |
| Yes | 50/290 | 275286(5.0) | 1 | 6.60×10^-3^ | 1 | **2.78×10^-2^** |
| No | 53/408 | 226687(4.2) | 0.464(0.267-0.808) |  | 0.557(0.331-0.938) |  |
| **Age ≥65** |  |  |  |  |  |  |
| ALI |  |  |  |  |  |  |
| Low | 389/698 | 1710013(28.5) | 1 | 2.67×10^-8^ | 1 | **4.66×10^-5^** |
| High | 196/512 | 834409(13.9) | 0.587(0.486-0.708) |  | 0.741(0.641-0.856) |  |
| Sleep duration(hours) |  |  |  |  |  |  |
| <7 & >9 | 217/415 | 891588(14.9) | 1 | 1.42×10^-1^ | 1 | 7.72×10^-2^ |
| 7 to 9 | 368/795 | 1652833(27.5) | 0.855(0.693-1.053) |  | 0.821(0.660-1.022) |  |
| Sleep trouble |  |  |  |  |  |  |
| Yes | 178/364 | 796694(13.3) | 1 | 8.89×10^-1^ | 1 | **1.26×10^-2^** |
| No | 407/846 | 1747727(29.1) | 1.017(0.801-1.289) |  | 0.773(0.632-0.946) |  |
|  |  |  |  |  |  |  |
| **BMI < 25** |  |  |  |  |  |  |
| ALI |  |  |  |  |  |  |
| Low | 181/359 | 832735(24.7) | 1 | 2.67×10^-8^ | 1 | **8.11×10^-4^** |
| High | 47/178 | 215825(6.4) | 0.599(0.418-0.859) |  | 0.581(0.422-0.798) |  |
| Sleep duration(hours) |  |  |  |  |  |  |
| <7 & >9 | 85/191 | 399391(11.8) | 1 | 8.92×10^-3^ | 1 | 1.40×10^-1^ |
| 7 to 9 | 143/346 | 649170(19.3) | 0.673(0.501-0.905) |  | 0.763(0.533-1.093) |  |
| Sleep trouble |  |  |  |  |  |  |
| Yes | 68/185 | 363119(10.8) | 1 | 4.21×10^-1^ | 1 | 4.98×10^-1^ |
| No | 160/352 | 685441(20.3) | 1.017(0.579-1.256) |  | 0.872(0.604-1.259) |  |
| **BMI 25 to 30** |  |  |  |  |  |  |
| ALI |  |  |  |  |  |  |
| Low | 167/369 | 742394(18.2) | 1 | 1.27×10^-2^ | 1 | **4.16×10^-4^** |
| High | 82/313 | 358721(8.8) | 0.672(0.492-0.918) |  | 0.568(0.715-0.777) |  |
| Sleep duration(hours) |  |  |  |  |  |  |
| <7 & >9 | 106/254 | 406756(10) | 1 | 1.33×10^-1^ | 1 | 1.45×10^-1^ |
| 7 to 9 | 143/428 | 694360(17) | 0.814(0.623-1.065) |  | 0.802(0.597-1.079) |  |
| Sleep trouble |  |  |  |  |  |  |
| Yes | 75/197 | 349023(8.6) | 1 | 8.46×10^-3^ | 1 | **1.38×10^-3^** |
| No | 174/485 | 752093(18.4) | 0.649(0.470-0.895) |  | 0.581(0.417-0.810) |  |
| **BMI ≥ 30** |  |  |  |  |  |  |
| ALI |  |  |  |  |  |  |
| Low | 92/223 | 385130(9.6) | 1 | 1.47×10^-1^ | 1 | **2.29×10^-2^** |
| High | 119/466 | 511588(12.8) | 0.789(0.574-1.086) |  | 0.691(0.502-0.950) |  |
| Sleep duration(hours) |  |  |  |  |  |  |
| <7 & >9 | 91/280 | 377971(9.4) | 1 | 7.55×10^-3^ | 1 | **4.50×10^-2^** |
| 7 to 9 | 120/409 | 518747(13) | 0.603(0.416-0.874) |  | 0.681(0.468-0.992) |  |
| Sleep trouble |  |  |  |  |  |  |
| Yes | 85/272 | 359838(9) | 1 | 3.00×10^-1^ | 1 | 4.47×10^-1^ |
| No | 126/417 | 536880(13.4) | 0.845(0.615-1.162) |  | 0.880(0.633-1.224) |  |
|  |  |  |  |  |  |  |
| **Age of cancer diagnosis < 40** |  |  |  |  |  |  |
| ALI |  |  |  |  |  |  |
| Low | 30/108 | 149274(6.7) | 1 | 9.79×10^-2^ | 1 | 1.35×10^-1^ |
| High | 26/183 | 117115(5.2) | 0.572(0.295-1.108) |  | 0.638(0.334-1.216) |  |
| Sleep duration(hours) |  |  |  |  |  |  |
| <7 & >9 | 28/121 | 129639(5.8) | 1 | 1.76×10^-1^ | 1 | 3.76×10^-1^ |
| 7 to 9 | 28/170 | 136751(6.1) | 0.621(0.312-1.238) |  | 0.813(0.386-1.711) |  |
| Sleep trouble |  |  |  |  |  |  |
| Yes | 27/133 | 126308(5.6) | 1 | 6.70×10^-3^ | 1 | **4.07×10^-2^** |
| No | 29/158 | 140081(6.2) | 0.465(0.268-0.808) |  | 0.536(0.295-0.974) |  |
| **Age of cancer diagnosis 40 to 60** |  |  |  |  |  |  |
| ALI |  |  |  |  |  |  |
| Low | 128/368 | 602311(11) | 1 | 1.55×10^-2^ | 1 | **2.18×10^-2^** |
| High | 79/442 | 361079(6.6) | 0.620(0.422-0.913) |  | 0.643(0.441-0.938) |  |
| Sleep duration(hours) |  |  |  |  |  |  |
| <7 & >9 | 104/322 | 439753(8.1) | 1 | 1.40×10^-3^ | 1 | **9.34×10^-3^** |
| 7 to 9 | 103/488 | 523637(9.6) | 0.535(0.364-0.785) |  | 0.582(0.387-0.875) |  |
| Sleep trouble |  |  |  |  |  |  |
| Yes | 88/299 | 444804(8.2) | 1 | 2.02×10^-2^ | 1 | **3.04×10^-2^** |
| No | 119/511 | 518586(9.5) | 0.611(0.403-0.925) |  | 0.641(0.429-0.959) |  |
| **Age of cancer diagnosis > 60** |  |  |  |  |  |  |
| ALI |  |  |  |  |  |  |
| Low | 143/332 | 607941(16.2) | 1 | 7.28×10^-2^ | 1 | 8.34×10^-2^ |
| High | 282/475 | 1208674(32.2) | 0.826(0.671-1.018) |  | 0.830(0.675-1.021) |  |
| Sleep duration(hours) |  |  |  |  |  |  |
| <7 & >9 | 150/282 | 614725(16.4) | 1 | 2.02×10^-2^ | 1 | 1.20×10^-1^ |
| 7 to 9 | 275/525 | 1201890(32) | 0.783(0.638-0.962) |  | 0.822(0.658-1.027) |  |
| Sleep trouble |  |  |  |  |  |  |
| Yes | 113/222 | 500868(13.3) | 1 | 1.11×10^-1^ | 1 | 1.26×10^-1^ |
| No | 312/585 | 1315747(35) | 0.814(0.633-1.048) |  | 0.824(0.645-1.053) |  |
| Abbreviations: ALI: advanced lung cancer inflammation index ; NHANES, the National Health and Nutrition Examination Survey; BMI: body mass index | | | | | |  |
| a Adjusted for age and/or gender(male/female). | | | | | |  |
| b Multivariable model additionally adjusted for race and ethnicity(non-Hispanic Black, Hispanic, non-Hispanic White, other), educational attainment(<high school, high school, >high school), family poverty income ratio(<1.3, 1.3 to ≤ 3.5, ≥3.5) and age of cancer diagnosis(<40, 40 to 60, >60). | | | | | |  |

| **eTable 3. Association of advanced lung cancer inflammation index and sleep quality with all-cause mortality among US cancer survivors age 40 years or older by cancer type, NHANES, 2005 to 2018.** | | | | | | |
| --- | --- | --- | --- | --- | --- | --- |
| **Mortality outcome** | **Death/No.** | **Weighted death(%)** | **Hazard ratio(95 % CI)** | | |  |
|  |  |  | **Model 1**a | **P** | **Model 2**b | **P** |
| **Digestive cancer** |  |  |  |  |  |  |
| ALI |  |  |  |  |  |  |
| Low | 51/80 | 203745(27) | 1 |  | 1 |  |
| High | 30/81 | 120560(16) | 0.604(0.316-1.154) | 1.27×10^-1^ | **0.540(0.295-0.990)** | **4.62×10**-2 |
| Sleep duration(hours) |  |  |  |  |  |  |
| <7 & >9 | 41/77 | 156129(20.7) | 1 |  | 1 |  |
| 7 to 9 | 40/84 | 168176(22.3) | 0.648(0.336-1.250) | 1.96×10^-1^ | 0.877(0.510-1.510) | 6.37×10^-1^ |
| Sleep trouble |  |  |  |  |  |  |
| Yes | 32/60 | 147594(19.6) | 1 |  | 1 |  |
| No | 49/101 | 176711(23.4) | 0.608(0.363-1.021) | 5.98×10^-2^ | **0.531(0.301-0.938)** | **2.93×10**-2 |
| **Genitourinary cancer** |  |  |  |  |  |  |
| ALI |  |  |  |  |  |  |
| Low | 123/278 | 458794(16.4) | 1 |  | 1 |  |
| High | 80/308 | 273929(9.8) | 0.816(0.553-1.203) | 3.00×10^-1^ | **0.631(0.437-0.911)** | **1.40×10**-2 |
| Sleep duration(hours) |  |  |  |  |  |  |
| <7 & >9 | 83/232 | 271875(9.7) | 1 |  | 1 |  |
| 7 to 9 | 120/354 | 460848(16.5) | 0.781(0.538-1.133) | 1.92×10^-1^ | 0.822(0.552-1.223) | 3.33×10^-1^ |
| Sleep trouble |  |  |  |  |  |  |
| Yes | 143/401 | 489799(17.5) | 1 |  | 1 |  |
| No | 60/185 | 242924(8.7) | 0.757(0.489-1.172) | 2.12×10^-1^ | 0.678(0.441-1.042) | 7.64×10^-2^ |
| **Skin cancer** |  |  |  |  |  |  |
| ALI |  |  |  |  |  |  |
| Low | 132/297 | 672065(14.7) | 1 |  | 1 |  |
| High | 68/297 | 345841(7.6) | 0.689(0.511–0.929) | 1.46×10^-2^ | **0.547(0.397–0.754)** | **2.22×10**-4 |
| Sleep duration(hours) |  |  |  |  |  |  |
| <7 & >9 | 78/206 | 408354(8.9) | 1 |  | 1 |  |
| 7 to 9 | 122/388 | 609553(13.4) | 0.540(0.401–0.726) | 4.72×10^-5^ | **0.563(0.416–0.762)** | **2.04×10**-4 |
| Sleep trouble |  |  |  |  |  |  |
| Yes | 64/207 | 350607(7.7) | 1 |  | 1 |  |
| No | 136/387 | 667299(14.6) | 0.801(0.532–1.205) | 2.86×10^-1^ | 0751(0.512–1.100) | 1.41×10^-1^ |
| **Breast cancer** |  |  |  |  |  |  |
| ALI |  |  |  |  |  |  |
| Low | 62/153 | 280288(16.4) | 1 |  | 1 |  |
| High | 39/154 | 195040(11.4) | 0.929(0.593–1.455) | 7.38×10^-1^ | 0.939(0.533–1.654) | 8.28×10^-1^ |
| Sleep duration(hours) |  |  |  |  |  |  |
| <7 & >9 | 40/116 | 173878(10.2) | 1 |  | 1 |  |
| 7 to 9 | 61/191 | 301450(17.7) | 0.840(0.517–1.363) | 4.80×10^-1^ | 0.639(0.401–1.018) | 5.94×10^-2^ |
| Sleep trouble |  |  |  |  |  |  |
| Yes | 73/204 | 357869(21) | 1 |  | 1 |  |
| No | 28/103 | 117458(6.9) | 1.442(0.834–2.494) | 1.90×10^-1^ | 1.703(0.985–2.945) | 5.66×10^-2^ |
| **Other cancer** |  |  |  |  |  |  |
| ALI |  |  |  |  |  |  |
| Low | 71/145 | 342149(21) | 1 |  | 1 |  |
| High | 31/114 | 150766(9.2) | 0.541(0.318-0.922) | 2.38×10^-2^ | 0.670(0.434-1.034) | 7.08×10^-2^ |
| Sleep duration(hours) |  |  |  |  |  |  |
| <7 & >9 | 40/94 | 173881(10.6) | 1 |  | 1 |  |
| 7 to 9 | 62/165 | 319033(19.5) | 1.051(0.671-1.645) | 8.28×10^-1^ | 0.836(0.514-1.360) | 4.72×10^-1^ |
| Sleep trouble |  |  |  |  |  |  |
| Yes | 44/99 | 213397(13.1) | 1 |  | 1 |  |
| No | 58/160 | 279517(17.1) | 0.493(0.287-0.846) | 1.03×10^-2^ | **0.530(0.306-0.918)** | **2.34×10**-2 |
| Abbreviations: ALI: advanced lung cancer inflammation index ; NHANES, the National Health and Nutrition Examination Survey; | | | | | | |
| a Adjusted for age and gender(male/female). | | | | | | |
| b Multivariable model additionally adjusted for race and ethnicity(non-Hispanic Black, Hispanic, non-Hispanic White, other), educational attainment(<high school, high school, >high school), family poverty income ratio(<1.3, 1.3 to ≤ 3.5, ≥3.5) and age of cancer diagnosis(<40, 40 to 60, >60). | | | | | | |

| **eTable 4. Joint association of advanced lung cancer inflammation index and sleep quality with all-cause, cancer, and non-cancer mortality among US cancersurvivors age 40 years or older, NHANES, 2005 to 2018.** | | | | | | | | |
| --- | --- | --- | --- | --- | --- | --- | --- | --- |
| **Mortality outcome** | **ALI** | **Death/No.** | **Weighted death(%)** | **Model 1^a^** | | **Model 2^b^** | | **P for  heterogeneity** |
|  |  |  |  | **HR(95%CI)** | **P-value** | **HR(95%CI)** | **P-value** |  |
| **All-cause** |  |  |  |  |  |  |  |  |
| Sleep duration <7 & >9 | Low | 177/351 | 751776(6.6) | 1 |  | 1 |  | 1.99×10^-2^ |
|  | High | 105/374 | 432341(3.8) | 0.669(0.500-0.895) | 6.78×10^-3^ | 0.600(0.451-0.791) | 4.34×10^-4^ |  |
| Sleep duration 7 to 9 | Low | 263/603 | 1208484(10.6) | 0.674(0.538-0.844) | 3.78×10^-8^ | 0.749(0.565-0.994) | 4.49×10^-2^ |  |
|  | High | 143/580 | 653793(5.7) | **0.491(0.381-0.633)** | **6.06×10^-4^** | **0.468(0.352-0.622)** | **1.72×10^-7^** |  |
| Sleep trouble Yes | Low | 145/304 | 696697(6.1) | 1 |  | 1 |  | 8.68×10^-3^ |
|  | High | 83/305 | 375283(3.3) | 0.597(0.444-0.804) | 6.66×10^-4^ | 0.462(0.339-0.628) | 8.08×10^-7^ |  |
| No | Low | 295/650 | 1263563(11) | 0.714(0.549-0.929) | 1.30×10^-2^ | 0.628(0.501-0.787) | 5.28×10^-5^ |  |
|  | High | 165/604 | 710851(6.2) | **0.553(0.406-0.755)** | **1.63×10^-4^** | **0.444(0.345-0.570)** | **2.18×10^-10^** |  |
| **Cancer** |  |  |  |  |  |  |  |  |
| Sleep duration <7 & >9 | Low | 50/351 | 218774(1.9) | 1 |  | 1 |  | 2.58×10^-1^ |
|  | High | 42/374 | 157457(1.4) | 0.782(0.488-1.253) | 3.07×10^-1^ | 0.714(0.438-1.163) | 1.76×10^-1^ |  |
| Sleep duration 7 to 9 | Low | 76/603 | 384543(3.4) | 0.773(0.513-1.165) | 2.19×10^-1^ | 0.548(0.546-1.370) | 5.36×10^-1^ |  |
|  | High | 52/580 | 234776(2) | **0.558(0.362-0.861)** | **8.40×10^-3^** | **0.865(0.347-0.867)** | **1.02×10^-2^** |  |
| Sleep trouble Yes | Low | 44/304 | 223950(2) | 1 |  | 1 |  | 4.33×10^-1^ |
|  | High | 31/350 | 110216(1) | 0.484(0.289-0.811) | 5.92×10^-3^ | 0.402(0.241-0.845) | 5.11×10^-4^ |  |
| No | Low | 82/650 | 379367(3.3) | 0.683(0.420-1.111) | 1.25×10^-1^ | 0.631(0.385-1.035) | 6.80×10^-2^ |  |
|  | High | 63/604 | 282018(2.5) | **0.627(0.389-1.012)** | **5.60×10^-2^** | **0.531(0.333-0.672)** | **7.59×10^-3^** |  |
| **Non-cancer** |  |  |  |  |  |  |  |  |
| Sleep duration <7 & >9 | Low | 127/351 | 533002(4.7) | 1 |  | 1 |  | 1.50×10^-2^ |
|  | High | 63/374 | 274884(2.4) | 0.623(0.451-0.862) | 4.15×10^-3^ | 0.555(0.410-0.751) | 1.38×10^-4^ |  |
| Sleep duration 7 to 9 | Low | 187/603 | 823941(7.2) | 0.634(0.489-0.822) | 6.18×10^-4^ | 0.704(0.518-0.955) | 2.43×10^-2^ |  |
|  | High | 91/580 | 419018(3.7) | **0.464(0.343-0.629)** | **6.84×10^-7^** | **0.440(0.315-0.615)** | **1.57×10^-6^** |  |
| Sleep trouble Yes | Low | 101/304 | 472746(4.1) | 1 |  | 1 |  | 2.86×10^-3^ |
|  | High | 52/350 | 265068(2.3) | 0.665(0.468-0.945) | 2.27×10^-2^ | 0.496(0.339-0.724) | 2.80×10^-4^ |  |
| No | Low | 213/650 | 884196(7.7) | 0.730(0.532-1.002) | 5.32×10^-2^ | 0.624(0.481-0.809) | 3.83×10^-4^ |  |
|  | High | 102/604 | 428834(3.7) | **0.514(0.364-0.726)** | **1.48×10^-4^** | **0.403(0.303-0.537)** | **5.68×10^-10^** |  |

| Abbreviations: ALI: advanced lung cancer inflammation index ; NHANES, the National Health and Nutrition Examination Survey; |
| --- |
| a Adjusted for age and gender(male/female). |
| b Multivariable model additionally adjusted for race and ethnicity(non-Hispanic Black, Hispanic, non-Hispanic White, other), educational attainment(<high school, high school, >high school), family poverty income ratio(<1.3, 1.3 to ≤ 3.5, ≥3.5) and age of cancer diagnosis. |
