# Supplementary figures and images for: Combined effects of nutrition, inflammatory status, and sleep quality on mortality in cancer survivors
Source: BMC Cancer. 2024 Nov 27;24:1456. doi: 10.1186/s12885-024-13181-x (PMC11600600; doi:10.1186/s12885-024-13181-x)

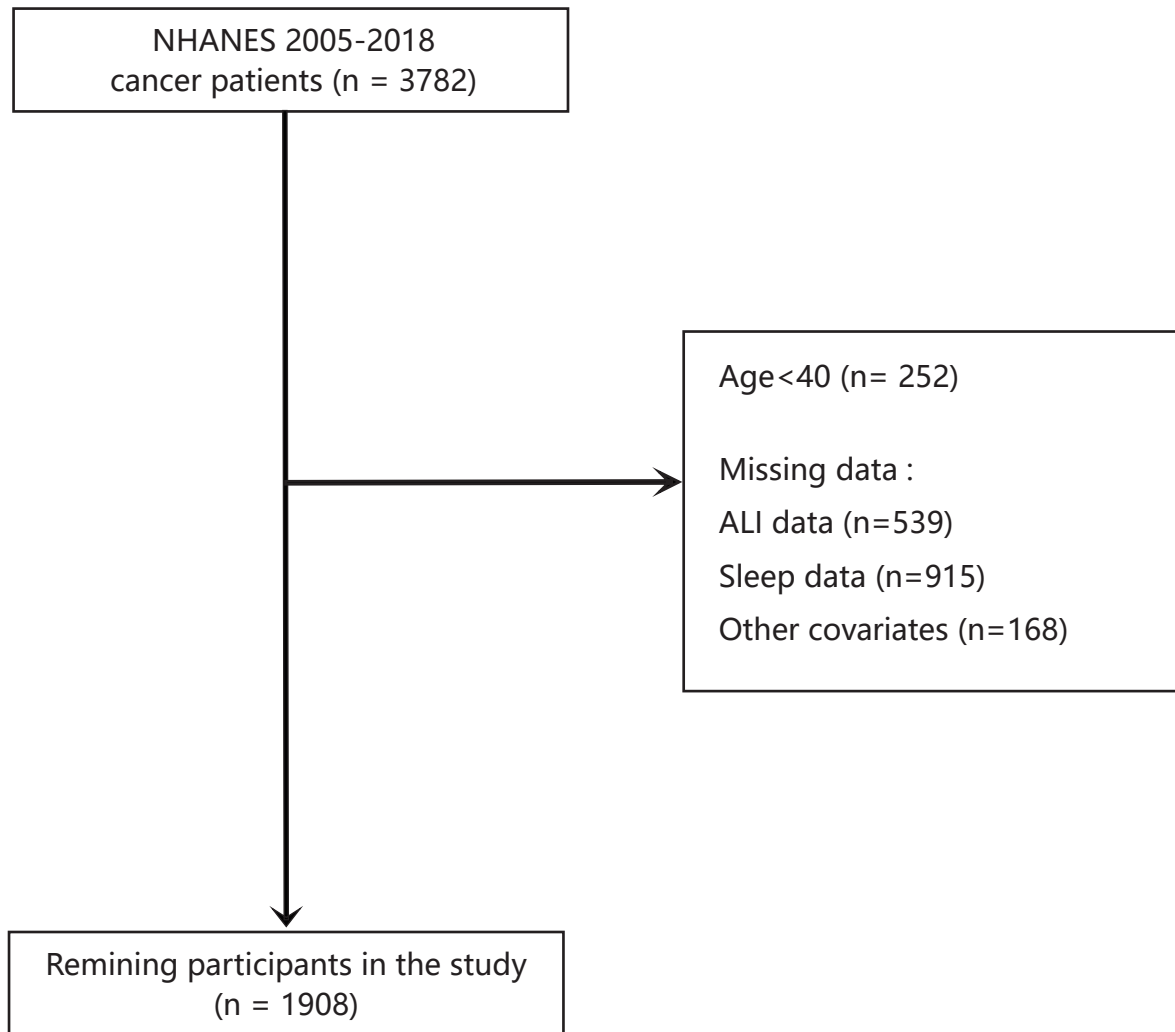

**eFigure 1.** Flowchart portraying the sample selection.

Supplement: Supplementary file 2 — Supplementary Material 2. [file 12885_2024_13181_MOESM2_ESM.pdf]
