# Supplementary material for: Combined effects of nutrition, inflammatory status, and sleep quality on mortality in cancer survivors
Source: BMC Cancer. 2024 Nov 27;24:1456. doi: 10.1186/s12885-024-13181-x (PMC11600600; doi:10.1186/s12885-024-13181-x)
Supplement: Supplementary file 3 — Supplementary Material 3. [file 12885_2024_13181_MOESM3_ESM.pdf]

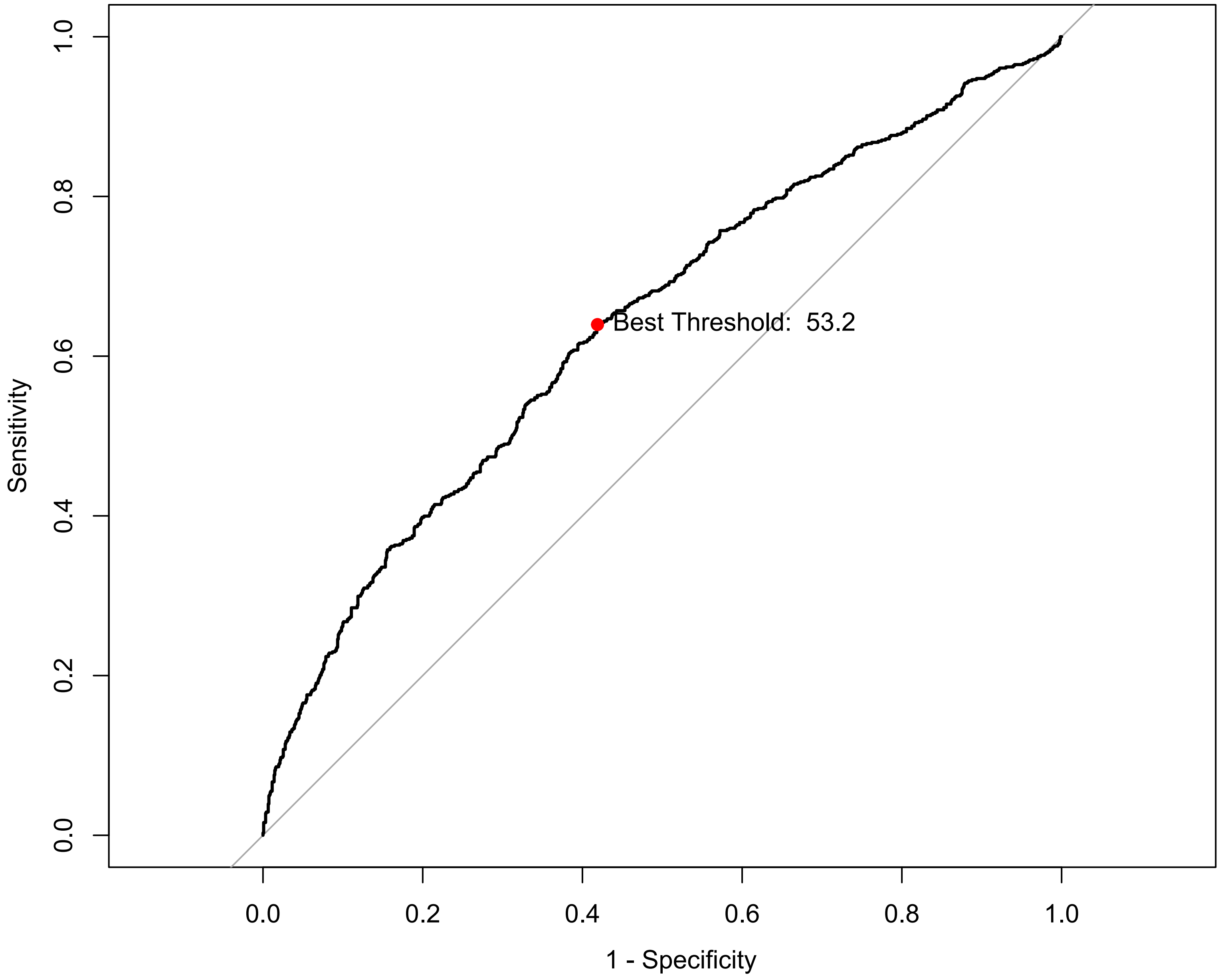

eFigure 2. Receiver operating characteristic curve determining the optimal cut-off value of advanced lung cancer inflammatory index.
