# Supplementary material for: Combined effects of nutrition, inflammatory status, and sleep quality on mortality in cancer survivors
Source: BMC Cancer. 2024 Nov 27;24:1456. doi: 10.1186/s12885-024-13181-x (PMC11600600; doi:10.1186/s12885-024-13181-x)
Supplement: Supplementary file 4 — Supplementary Material 4. [file 12885_2024_13181_MOESM4_ESM.pdf]

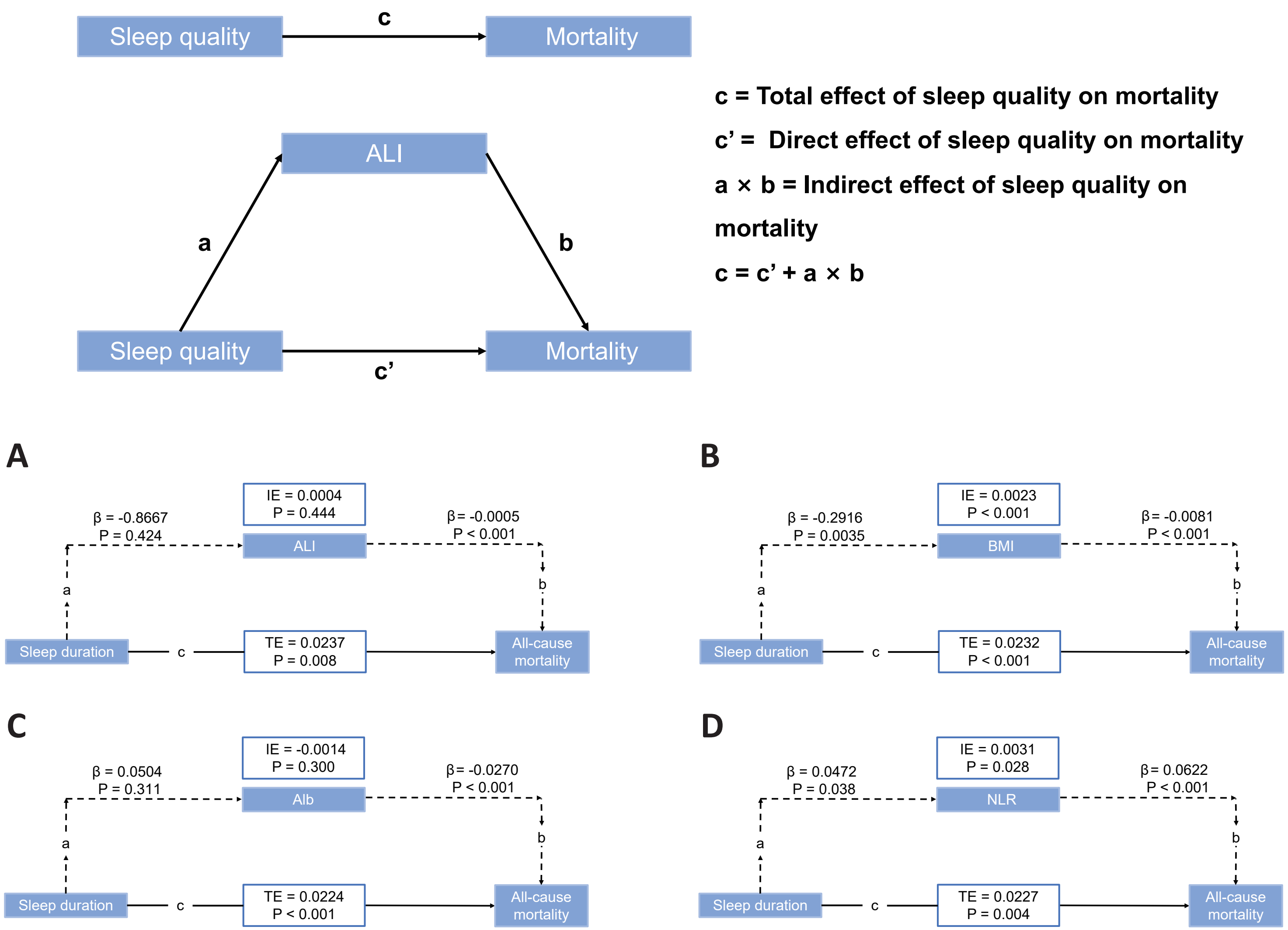

eFigure 3. Mediation effect analysis of the association between nutrition, iInflammatory status, sleep quality, and mortality outcomes. (A) Adcanced lung cancer inflammatory index; (B) Body mass index; (C) Serum albumin; (D) Neutrophil-to-lymphocyte ratio. IE: Indirect effect; TE: Total effect.
